# *NONO*‐Related Syndromic X‐Linked Developmental Disability 34: Further Clinical and Molecular Delineation in a Prenatal Cohort

**DOI:** 10.1002/pd.70097

**Published:** 2026-02-18

**Authors:** Yilin Zhao, Gang Zou, Mengmeng Shi, Kwok Ming Law, So Ling Lau, Meng Meng, Yuen Ha Ting, Chiu Yee Liona Poon, Zirui Dong, Matthew Hoi Kin Chau, Tak Yeung Leung, Kwong Wai Choy, Xinwen Zhang, Ye Cao

**Affiliations:** ^1^ Reproductive Medicine Institute Xi'an People's Hospital (Xi'an Fourth Hospital) Xi'an China; ^2^ Department of Obstetrics and Gynaecology The Chinese University of Hong Kong Hong Kong SAR China; ^3^ Shanghai Key Laboratory of Maternal Fetal Medicine Department of Obstetrics Fetal Medicine Unit and Prenatal Diagnosis Center Shanghai First Maternity and Infant Hospital Tongji University School of Medicine Shanghai China; ^4^ Shenzhen Research Institute The Chinese University of Hong Kong Shenzhen China; ^5^ The Chinese University of Hong Kong‐Baylor College of Medicine Joint Center for Medical Genetics Hong Kong SAR China

**Keywords:** genome sequencing, intellectual disability, IUGR, MRXS34, non‐compaction cardiomyopathy, *NONO,* corpus callosum anomalies, syndromic X‐linked intellectual disability 34

## Abstract

**Objective:**

To characterize the prenatal sonographic features across different trimesters and genomic spectrum of *NONO*‐related X‐linked intellectual developmental disorder.

**Method:**

We analyzed two fetuses presenting with corpus callosum agenesis and rare cardiac anomalies using genome sequencing and exome sequencing. A systematic literature review was conducted to provide a comprehensive analysis of genotype‐phenotype correlations.

**Results:**

Two novel cases were reported in this study: one with a de novo 61.7 kb deletion affecting both *NONO* and *ITGB1BP2*, and another with a de novo c.1093 C > T(p.Arg365Ter), a recurrent variant reported in the literature. A total of 23 cases with *NONO* defects and prenatal phenotypes were identified, encompassing 19 distinct variant types. Large deletions and splicing variants accounted for approximately 32% (6/19). Among all cases, 78% (18/23) exhibited typical or concurrent brain abnormalities, such as corpus callosum agenesis, cardiac defects including left ventricular noncompaction (LVNC), or short long bones in the second trimester. Notably, 4% (1/23) of cases were reported with isolated intrauterine growth restriction (IUGR), primarily identified in the third trimester.

**Conclusion:**

Genome sequencing facilitates thorough identification of the genetic causes of NONO‐related syndrome. The recurrent variant p.Arg365Ter has been reported to cause variable cardiac abnormalities in different patients, suggesting that other genetic or non‐genetic factors may contribute to the cardiac manifestations in NONO‐related syndromes.

## Key Points

- What is already known about this topic?
  ◦ Hemizygous loss‐of‐function variants in the *NONO* gene cause X‐linked syndromic intellectual developmental disorder‐34 (MRXS34).
  ◦ Prenatal ultrasound features of *NONO*‐related syndrome have been described only to a limited extent, and the associated phenotypes are known to be variable.
- What does this study add?
  ◦ NONO‐related syndrome typically shows no distinct features in the first trimester. However, by the second trimester, 86% of cases exhibit typical or concurrent brain abnormalities, such as agenesis of the corpus callosum, cardiac issues like left ventricular noncompaction (LVNC), or short long bones. Some cases exhibit intrauterine growth restriction (IUGR) solely in the third trimester without other structural anomalies. In rare cases, no prenatal abnormalities are observed at all.
  ◦ Large deletions and splicing variants were commonly reported in NONO‐related syndromes.
  ◦ Genome sequencing offers comprehensive detection of genomic variations, facilitating the thorough identification of the genetic causes of NONO‐related syndrome.

## 1 Introduction

The *NONO* gene, located on chromosome Xq13.1, encodes a protein belonging to the highly conserved *Drosophila* behavior/human splicing (DBHS) protein family, which has been characterized as an important transcriptional regulator in diverse cellular contexts [1]. Hemizygous loss‐of‐function variants in the *NONO* gene were first reported in two male patients with intellectual disability, macrocephaly, thick corpus callosum, and cerebellum hypoplasia in 2015 [2]. Defects in *NONO* cause a neurodevelopmental disorder, known as syndromic X‐linked intellectual disability 34 (MONDO:0,010,501, also referred to as MRXS34; OMIM: 300967). Subsequent studies have described additional cardiac anomalies in patients with MRXS34, including left ventricular noncompaction cardiomyopathy (LVNC), septal defects, and pulmonary stenosis, in patients with MRXS34. Postnatal features typically include global developmental delay, intellectual disability, corpus callosum anomalies, and relative macrocephaly [3]. To date, over 30 patients with pathogenic variants in *NONO* have been reported, with around 20 cases having prenatal phenotypes reported with variable features of cardiomyopathy and central neural system anomalies, predominantly detected by ultrasound between 17–26 gestational weeks (GW) [3, 4, 5, 6, 7, 8, 9, 10]. Nevertheless, further cases are needed to better delineate the prenatal profile in different trimesters associated with MRXS34 for improved diagnosis.

Here we report two male fetuses, both presenting with agenesis of corpus callosum and cardiac abnormalities, including LVNC and displacement of the tricuspid valve. These cases were identified with loss‐of‐function variants in the *NONO* gene, which are expected to result in haploinsufficiency and reduced *NONO* gene expression. Our report contributes to the expanding phenotypic spectrum associated with *NONO* gene defects. Furthermore, we reviewed reported cases with prenatal phenotype, to better delineate prenatal genotype‐phenotype correlations of this syndrome.

## 2 Methods

This study was conducted retrospectively, focusing on cases diagnosed with *NONO* gene defects in our centers between Mar 2021 and June 2025. Fetal DNA was extracted from direct amniotic fluid samples for genetic testing. Parental peripheral blood was also collected for DNA extraction to allow trio‐based analyses. Chromosomal microarray analysis (CMA) was performed using FetalDNA Chip v2.0 (8 × 60 K, in‐house designed aCGH + SNP array, Agilent Technologies, https://www.obg.cuhk.edu.hk/services/laboratory‐services/chromosomal‐microarray‐analysis/) [11, 12]. Fetal genome sequencing (minimal 30X read depth) or trio exome sequencing (mean coverage 200x, with over 98% of target bases achieving at least 30x) approach and data interpretation were according to the previously published studies, following the published Protocols [23, 24]. The detected genomic variants were interpreted and reported according to the American College of Medical Genetics and Genomics (ACMG) guidelines. The medical records of patients were meticulously collected and reviewed. This study received approval from our institutional Research Ethics Board (CREC no.2016.713). Informed written consent was obtained from the participants involved.

### 2.1 Literature Review

Literature search used the MESH terms: “((1966/01/01:2025/12/15[Date ‐ Publication] AND (NONO [All Fields]) AND ((((intellectual disability) OR (agenesis of corpus callosum)) OR (heart defects, congenital)) OR (prenatal)))” in PubMed and we also searched in Google scholar. A total of 36 publications were extracted from PubMed, with one additional publication from Google Scholar. After thorough review, we included 11 publications that contained 21 prenatal cases with confirmed pathogenic *NONO* variants and available prenatal findings in our study. Including the two cases in this study, 23 cases of NONO‐related syndrome are summarized in Table 1, which includes prenatal phenotypes in different trimesters and pregnancy outcomes [2, 4, 5, 8, 9, 10, 13, 14, 15, 16, 17]. The complexity of the disorder is evident in the variability of phenotypes and genetic spectrum among patients.

**TABLE 1:** Clinical and genetic summary of the *NONO* variants in the prenatal period.

Study.	*NONO* variant (NM_001145408) and inheritance		First trimester	Second trimester	Third trimester	Pregnancy outcome and recent estimate age	Other genetic findings
Mircsof et al. (2015) [2]	1	c.1394dup; p.Asn466LysfsTer13, maternally inherited	—	Hydramnios and short long bones	—	Livebirth and 15‐year‐old	—
	2	c.1093 C>*T*; p.Arg365Ter	—	—	IUGR at 38 GW	Livebirth and 20‐year‐old	—
Carlston et al. (2019) [8]	3	c.154 + 5_154+6delGT; p.Asn52SerfsTer6, de novo	—	Prenatal‐onset growth failure with preservation of head circumference (did not specific the GW)		Livebirth and 32 months	*TTN*:NM_001267550.2:c.36737 A>*T*, p.Glu12246Val, maternally inherited
Sewani et al. (2020) [9]	4	c.1171 + 1G>A, de novo	—	Polyhydramnios, short limbs and a brain abnormality at 20 GW	Polyhydramnios and macrocephaly	Livebirth, 4‐year old male	—
	5	c.457 C>*T*, p.Arg153Ter, de novo	—	—	—	Miscarried at 16 4/7 GW	—
Sun et al. (2020) [5]	6	c.154 + 9A>G, p.Asn52SerfsTer3, maternally inherited	—	Hypoplastic left heart syndrome at 26 GW	—	TOP	*MYH6*: NM_002471.3:c.718 G>A, p.Asp240Asn, paternally inherited
Sun et al. (2020b) [4]	7	c.246_249del, p.Pro83ThrfsTer7	—	Biventricular noncompaction, EA, PS, VSD, pericardial effusion	—	TOP	—
	8	c.246_249del, p.Pro83ThrfsTer7	—	LVNC, PA, VSD, aorta astride, right aortic arch, pulmonary dysplasia, transposition of the aorta, PLSVC	—	TOP	—
	9	c.246_249del, p.Pro83ThrfsTer7	—	LVNC, PS, VSD, right ventricular diverticulum, PLSVC	—	TOP	—
	10	c.471del, p.Gln157HisfsTer18	—	LVNC, ebstein anomaly, PS, ASD, variation of branch of aortic arch	—	TOP	—
	11	c.471del, p.Gln157HisfsTer18	—	Biventricular noncompaction, EA, PS, VSD	—	TOP	—
Roessler et al. (2022) [10]	12	c.217 C>*T*, p.Arg73Ter, de novo	—	Complex heart defects (hypertrabeculation, a mildly dysplastic pulmonary valve, multiple small VSDs and a right aortic arch) at 20 GW	Macrocephaly, agenesis of the corpus callosum and asymmetric IUGR with shortened long bones	Livebirth, 78‐days‐old	—
	13	c.1009 C>*T*, p.Arg337Ter, de novo	—	EA (did not specify the GW)		Livebirth and 6‐year‐old	—
	14	c.90_114del, p.Gln30HisfsTer18, de novo	—	Hypoplastic and partial agenesis of the corpus callosum and a mild cerebral ventriculomegaly (did not specify the GW)		Livebirth and 1‐year‐old	—
Giuffrida et al. (2022) [13]	15	arr[GRCh37] Xq13.1del (70,520,984–70536128) x1, 15.1kb	—	The absence of septum pellucidum, pericallosal artery with complete corpus callosum agenesis and cardiomegaly with wall hypertrophy and disproportion with a prevalence of the right heart chambers, wide tricuspid insufficiency and downward displacement of septal leaflet, suspecting EA at 22 GW. The other fetal structures appeared normal.	—	TOP	—
Writzl et al. (2023) [14]	16	c.348 + 2_ 348 + 15del, de novo	—	Hydronephrosis (did not specify the GW)		Livebirth, unexpectedly died at the age of 17	—
Rodriguez‐revenga et al. (2024) [15]	17	c.355 C>*T*, p.Arg119Ter	—	Right multicystic dysplastic kidney, left renal agenesis, absent urinary bladder, oligohydramnios, myocardial hypertrophy, increased nuchal fold (7.3 mm) and a single umbilical artery at 16 6/7 GW	—	TOP	—
Planté‐bordeneuve et al. (2025) [16]	18	c.279_282del, p. Phe94ArgfsTer27	—	Short long bones (did not specify the GW)		Livebirth and 3‐year‐old	—
	19	c.201_202dup, p.Lys68ArgfsTer24	—	Macrocephaly, short long bones (did not specific the GW)		Livebirth and 19‐year‐old	—
	20	c.710del, p.Pro237GlnfsTer6	—	Unremarkable pregnancy		Livebirth and 25‐year‐old	—
Huang et al. (2026) [17]	21	c.214 C>*T* p.Gln72Ter	—	Noncompaction cardiomyopathy, mild ventriculomegaly (10 mm/millimeters) and relative macrocephaly (97th percentile) at 23 GW; Mild ventriculomegaly (10.6 mm) and agenesis of the corpus callosum at 25 GW.	—	—	—
Case 1	22	seq[GRCh37] del(X) (q13.1)dn (70,479,500_70541202)x 1, 61.7kb	Nuchal translucency (NT) 2.4 mm), hypoplastic nasal bone, reversed ductus venosus a‐wave and tricuspid regurgitation	LVNC and dilated anterior horns of the lateral cerebral ventricles at 23 GW	Severe cardiomegaly (cardio‐thoracic ratio 75%), left ventricle showed severe hypo‐contractility, dysgenesis and increase in thickness of the corpus callosum (short length below 5th centile for GA)	Certified dead 2 hours after birth	46, XY, inv(9) (p12q13)
Case 2	23	c.1093 C>*T* p.Arg365Ter, de novo	—	Agenesis of the corpus callosum and displacement of the tricuspid valve	—	TOP at 26 GW	—
Total number	23	Variants: 19 Small variants: 17 ‐Splicing region: 4 ‐Coding region: 13 CNV: 2	0/23	21/23	2/11	TOP:9 Miscarriage:1 Neonatal death:1	
				Cardiac abnormalities: 13/23 Brain abnormalities: 7/23 Short long bones: 4/23 Polyhydramnios: 2/23 Nephric abnormalities: 2/23	IUGR/short long bone: 2		

Abbreviations: EA: Ebstein anomaly; GW: gestational week; IUGR: intrauterine growth retardation; LVNC: left ventricular non‐compaction; PA: pulmonary atresia; PLSVC: persistent left superior vena cava; PS: pulmonary stenosis; TOP: termination of pregnancy; VSD: ventricular septal defect; ‐: no data reported.

## 3 Results

### 3.1 Case 1

**FIGURE 1:**
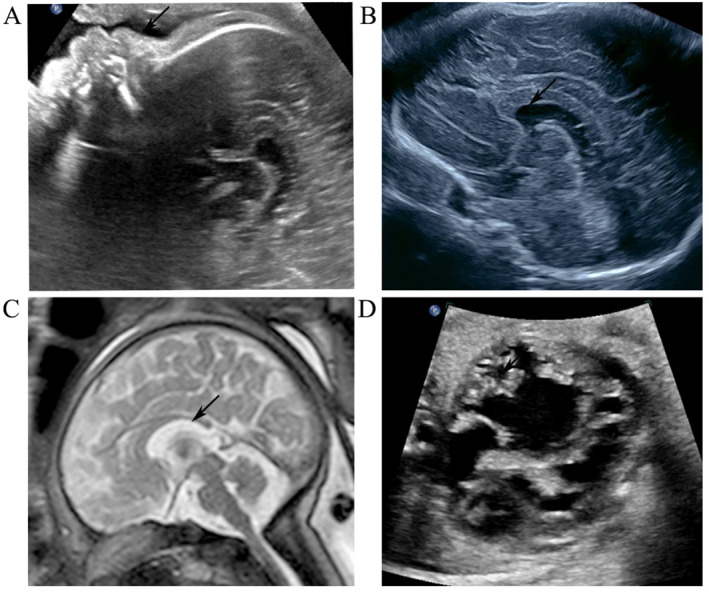
(A) Arrow indicates the hypoplastic nasal bone detected by the ultrasound. (B) The arrow indicates the dilated anterior horns of the lateral cerebral ventricles detected at the routine fetal morphology scan at 23 GW. (C) The arrow points to a short corpus callosum identified by fetal MRI. (D) Fetal ultrasound at 35 GW showed severe cardiomegaly, with globularly dilated left ventricle (LV). The arrow indicates prominent trabeculae.

The mother is a 28‐year‐old primigravida. Her first trimester combined screening test for Down syndrome at 12 gestational weeks was low risk with nuchal translucency (NT) of 2.4 mm. Other findings included hypoplastic nasal bone (Figure 1A), reversed ductus venosus a‐wave, and tricuspid regurgitation. A routine fetal morphology scan was performed at 23 GW, which suspected LVNC and dilated anterior horns of the lateral cerebral ventricles (Figure 1B). The head circumference (HC) was 2SD above the mean for gestational age (GA), and the other growth parameters were within normal ranges. Fetal MRI revealed a short corpus callosum (Figure 1C). Subsequent scans showed similar findings, but the HC progressively increased in size to more than 2 SD after 26 GW, whereas other parameters remained in normal ranges for GA.

The patient did not return for follow‐up in our center until 34 GW. Fetal ultrasound at 35 GW showed severe cardiomegaly (cardio‐thoracic ratio 75%) with globularly dilated left ventricle (LV) and bulging of the ventricular septum into the right ventricle (RV). The LV showed prominent trabeculae with deep intertrabecular recesses communicating with the LV cavity, which was compatible with LVNC (Figure 1D). The RV function was normal with mild myocardial thickening. Dysgenesis of the corpus callosum was noted with a short length below fifth centile for GA, a mild increase in thickness, and a deficient rostrum. No ventriculomegaly or other intracranial abnormality was observed. Fetal brain MRI confirmed the ultrasound findings. There was relative macrocephaly with head circumference > 3 SD above the mean for GA and frontal bossing. Plantar edema of both feet was also observed.

Given the severe LV failure and the high chance of neonatal death expected, the couple opted for palliative care without active resuscitation of the baby after counseling by a pediatric cardiologist and neonatologist. Induction of labor was performed at 38 GW to avoid cephalopelvic disproportion caused by macrocephaly. A male baby, 3.19 kg was born vaginally with Apgar scores 0 at 1, 5, and 10 min. The heart rate was 40–60 per minute immediately after birth, but was inaudible shortly afterward, with only pulseless electrical activity recorded by ECG. The baby was certified dead 2 hours after birth. The couple declined the post‐mortem of the baby.

**FIGURE 2:**
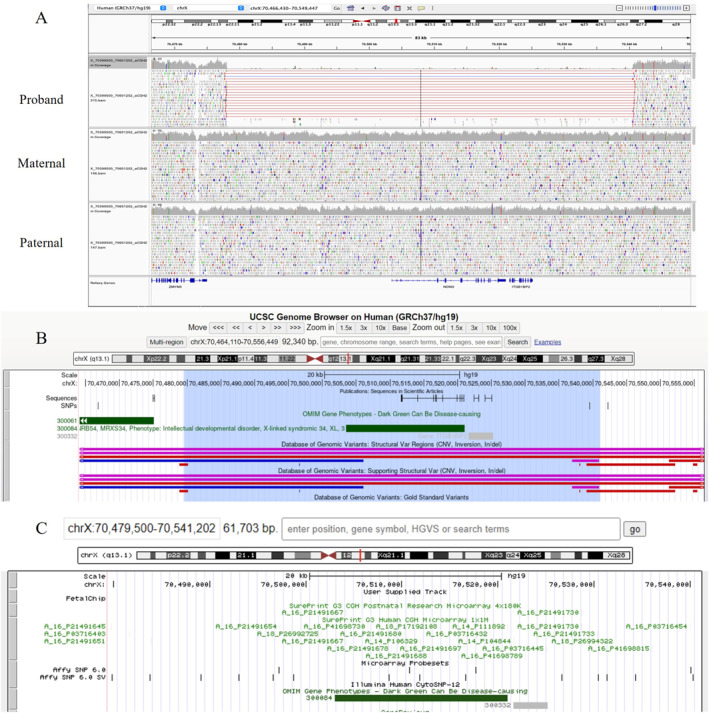
(A) The Integrative Genomics Viewer (IGV) displays the genome sequencing results of the de novo 61.7 kb deletion detected in the fetus, which was not identified in parental samples of Case 1. (B) The UCSC Genome Browser view shows deletion region (highlighted in blue), including annotations from the Database of Genomic Variants and OMIM database. (C) The UCSC Genome Browser view illustrates probe distribution across the entire target region and specifically within the *NONO* gene region on the FetalChip V2.0, as well as on other chromosome microarray or SNP detection platforms.

CMA analysis did not identify pathogenic copy number variants. Chromosome analysis detected 46, XY, inv(9)(p12q13) at 23 GW. Cord blood was collected, and trio‐based genome sequencing (GS) was performed for further investigations. The Trio GS identified a de novo hemizygous pathogenic copy number loss of chromosome band Xq13.1 of approximately 61.7 kb in size involving the whole *NONO* gene (ClinVar number: SCV006303306) (Figure 2A and 2B). Loss‐of‐function variants in the *NONO* gene are known to cause MRXS34 [MIM, 300,967], and haploinsufficiency of the *NONO* gene is a known disease mechanism with an HI Score of 3 (ClinGen Dosage ID:ISCA‐33548). Features of this disorder can include delayed psychomotor development, intellectual disability with poor speech, dysmorphic facial features, and mild structural brain abnormalities, including thickening of the corpus callosum [2, 6, 7]. This deletion was not detected by prenatal CMA due to the lack of probes in the relevant regions and beyond the CMA resolution (> 100kb) (Figure 2C). This deletion explained the prenatal features in this case.

### 3.2 Case 2

**FIGURE 3:**
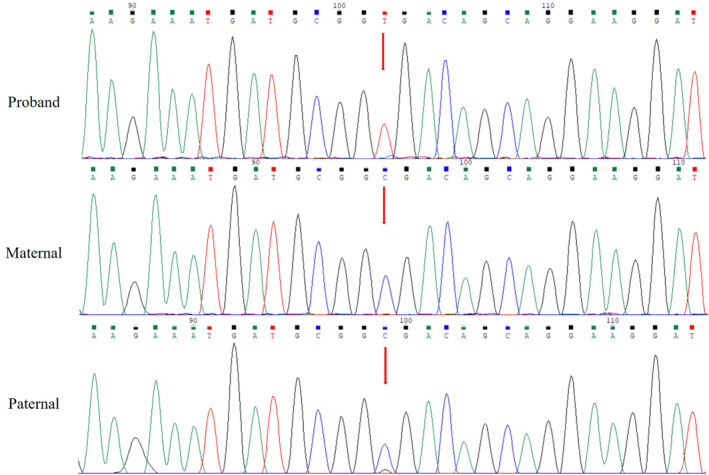
Sanger validation of the c.1093C>T variant in the *NONO* gene detected by the WES in Case 2. Red arrow indicates the de novo c.1093C>T variant identified in the fetus.

The patient is a 28‐year‐old Chinese primigravida with a natural pregnancy. Prenatal ultrasound in the second trimester revealed agenesis of the corpus callosum and displacement of the tricuspid valve. Amniocentesis was performed at 20 GW. CMA analysis did not identify pathogenic copy number variants, and chromosome analysis showed 46, XY. Trio exome sequencing detected a de novo heterozygous nonsense c.1093C>T (p.Arg365Ter) in the *NONO* gene in the fetus (NM_001145408.2). Sanger validation of this variant in this family was also performed (Figure 3). This known pathogenic variant has been reported in two postnatal cases [2]. Due to the poor prognosis, the parents chose to terminate the pregnancy at 26 GW.

### 3.3 Literature Review

As of 2025, we reviewed prenatal phenotypes in total 23 cases with defects in the *NONO* gene, including 21 published cases. Interestingly, although most variants are unique, some variants, such as c.1093C>T (p.Arg365Ter), are recurrent and de novo in these cases. Most of these variants are either nonsense or frameshift variants involving coding exons. Large deletions and splicing variants accounted for approximately 32% (6/19). Splicing variants, particularly those outside the canonical splice sites, are relatively common (3/19, 16%). None of the cases showed significant changes in the first‐trimester ultrasound. However, 83% (19/23) of cases demonstrated various structural abnormalities on the second‐trimester fetal scan, with the most typical and severe findings being cardiac abnormalities (52%, 12/23) or brain anomalies (30%, 7/23). If a molecular diagnosis is confirmed and structural abnormalities are severe, families are likely to opt for termination of pregnancy (TOP). For the 11 cases with available third‐trimester information, the phenotypes largely followed the patterns seen in the second trimester. Notably, intrauterine growth restriction (IUGR) was the primary indication for NONO‐related syndrome in the third trimester (1/11).

## 4 Discussion

This report describes two male fetuses with cardiac abnormalities and brain anomalies with defects in the *NONO* gene. Our cases, along with the literature review, underscore the concurrent cardiovascular and nervous system developmental abnormalities as key prenatal features of MRXS34.

Previous studies have suggested that central nervous system abnormalities such as agenesis of the corpus callosum (ACC) have rarely been reported prenatally and are not considered as the key prenatal presentation, while cardiovascular anomalies are the most common features in NONO‐defect cases, accounting for around 89.4% of the reported patients [3, 4, 10]. In contrast, our review found that brain abnormalities such as corpus callosum anomalies were detected prenatally in seven out of 23 cases (Table 1). The concurrent cardiovascular and nervous system developmental abnormalities suggested the *NONO* gene may play a role in an overlapping mechanism that allows functional crosstalk between the two systems. The link between cardiovascular and nervous system development has been increasingly recognized. Congenital heart defects (CHD) are consistently associated with a relatively high prevalence of structural brain abnormalities (43%) and a significant risk of neurodevelopmental delay, even in the absence of known chromosomal or genetic abnormalities [18]. The *NONO* gene encodes a protein that belongs to the highly conserved DBHS protein family and is broadly expressed in human tissues, including the brain, skeleton, and cardiovascular system [19]. Interestingly, NONO‐deficient mice were observed to have facial anomalies, a small cerebellum, and impaired cardiovascular function [20, 21]. However, it remains unclear why *NONO* variants primarily cause cardiovascular and nervous system developmental abnormalities, and further exploration of this variant is warranted.

The cardiovascular manifestations in *NONO*‐defect cases are highly variable, ranging from no symptoms to conditions such as left ventricular noncompaction (LVNC) and heart failure (Table 1). Variants in other cardiomyopathy‐related genes, such as *TTN* and *MYH6,* were also reported in patients with MRXS34 [5]. Cases with the same variant, such as p.Arg365Ter, showed different fetal cardiac abnormalities. The first case, from the DDD cohort, had no significant antenatal findings except late onset intrauterine growth restriction [2]. The second case was a 10‐year‐old Hispanic male who showed a small secundum atrial septal defect (ASD), a small apical muscular ventricular septal defect (VSD), a small patent ductus arteriosus (PDA) and right ventricular hypertrophy. LVNC was diagnosed at 1 month of age. His ASD, VSD and PDA closed spontaneously by 4 months of age. His left ventricular function has remained in the normal range, but continued follow‐up for LVNC is needed [7]. The third case corresponds to Case 2 in our study. Prenatal ultrasound at the second trimester revealed agenesis of the corpus callosum and displacement of the tricuspid valve. These findings suggest that the loss of *NONO* function may predispose patients to the development of CHD and LVNC with variable penetrance or expressivity influenced by other genetic or environmental modifiers. In our Case 1, a 61.7 kb deletion was detected, including the *NONO* gene and the *ITGB1BP2* gene. A previously published report described a fetus with a deletion with a minimum size of 15 kb encompassing 3′UTR (3 noncoding exons) of *NONO*, the whole *ITGB1BP2,* and an intronic sequence of *BCYRN1*, presenting with a similar cardiac phenotype to Case 1. The *ITGB1BP2* gene encodes a muscle‐specific signaling protein (Melusin), specifically expressed in heart and skeletal muscles. Four variants in the *ITGB1BP2* gene have been found so far in patients with hypertension or primary hypertrophic cardiomyopathy, suggesting a potential etiologic contribution of the *ITGB1BP2* gene to human cardiomyopathy [22]. These findings raise the possibility that *ITGB1BP2* contributes to the severity and variability of cardiac manifestations in patients with MRXS34.

According to our review, prenatal clinical diagnosis of MRXS34 is typically made in the second trimester, based on the presence of co‐occurring ACC and characteristic heart malformations, especially LVNC and tricuspid valve abnormalities (Table 1). Fetal abnormalities during the first trimester were neither specific nor reliably detectable for this syndrome. Various types of variants in the *NONO* gene have been reported; however, if CMA does not typically cover the *NONO* gene, this may lead to missed genetic diagnoses. This highlights the importance of genomic sequencing (GS) in the comprehensive and precise detection of genomic variations in the prenatal diagnosis of MRXS34. For fetuses with concurrent findings of ACC/other CNS anomalies and characteristic cardiac defects (especially LVNC), targeted analysis of the *NONO* gene or comprehensive sequencing is strongly indicated.

## Funding

This work was supported by Collaborative Research Fund (No.: C4062‐21 GF), General Research Fund (No.: 14,112,221), Natural Science Basic Research Plan in Shaanxi Province of China (Program No.: 2021JQ‐936), CUHK Direct Grant (2025.164) and (2024.157).

## Ethics Statement

The study was approved by the Joint Chinese University of Hong Kong – New Territories East Cluster Clinical Research Ethics Committee (CREC Ref. No.:2016.713).

## Consent

Informed patient consent was obtained for each case.

## Conflicts of Interest

The authors declare no conflicts of interest.

## Data Availability

The data that support the findings of this study are available on request from the corresponding author. The data are not publicly available due to privacy or ethical restrictions.
